# The complete chloroplast genome sequence of *Senna bicapsularis*

**DOI:** 10.1080/23802359.2020.1765216

**Published:** 2020-05-14

**Authors:** Zhe Hou

**Affiliations:** a Key Laboratory of Southwest China Wildlife Resources Conservation (Ministry of Education), China West Normal University, Sichuan, Nanchong, China; b State Key Laboratory of Tree Genetics and Breeding, Chinese Academy of Forestry, Beijing, China

**Keywords:** *S. bicapsularis*, chloroplast genome, phylogenetic analysis, genetic information

## Abstract

The complete chloroplast genome sequence of *Senna bicapsularis* was characterized from Illumina pair-end sequencing. The chloroplast genome of *S. bicapsularis* was 161,056 bp in length, containing a large single-copy region (LSC) of 90,416 bp, a small single-copy region (SSC) of 18,538 bp, and two inverted repeat (IR) regions of 26,051 bp. The overall GC content is 36.20%, while the correponding values of the LSC, SSC, and IR regions are 64.5%, 69.4%, and 60.2%, respectively. The genome contains 129 complete genes, including8 rRNAs, 37 tRNAs and 84 protein coding genes. A phylogenetic analysis showed that *Senna tora* and *Erythrophleumfordii*form the basis of the produced evolutionary tree. *S.bicapsularis* and *S. occidentalis*, which belongto the group *Cassia*, share the closest relationship. The analysis of the cp genome of *S.bicapsularis*provides crucial genetic information for further studies of this precious species and the taxonomy, phylogenetics and evolution of *Cassia*.

*Senna bicapsularis* (family: Fabaceae) is an ornamental plant belonging to *Cassia* species which is widely distributed in South American and tropical countries. Traditionally, plants belonging to *Cassia* species are believed to possess medicinal values. The wood of *S. bicapsularis* can be used to make paper pulp and its fruits are said to be edible (Mak et al. 2013). Nevertheless, its genetic background and resources have not been widely studied. Polymorphic chloroplast microsatellite markers designed based on a cp genome analysis can be utilized to comprehend the levels and patterns of the geographical structure and genetic diversity of *S. bicapsularis*, and this information can subsequently be used to formulate an effective protection strategy.

A single individual of *S. bicapsularis* was used as a sampling object from the China West Normal University (106°08′E; 30°78′N) in Nanchong. Fresh leaves of the individuals were collected and flash-frozen in liquid nitrogen and then stored in a refrigerator (–80 °C) until DNA extraction. The voucher specimen (SJJM001) was laid in the Herbarium of China West Normal University and the extracted DNA was stored in the –80 °C refrigerator of the Key Laboratory of Southwest China Wildlife Resources Conservation. We extracted total genomic DNA from 25 mg silica-gel-dried leaf using a modified CTAB method (Doyle 1987). The Illumina HiSeq 2000 platform (Illumina, San Diego, CA) was used to perform the genome sequence. We used the software MITObim 1.8 (Hahn et al. 2013) and metaSPAdes (Nurk et al. 2017) to assemble chloroplast genomes. We used *S. occidentalis* (GenBank: NC_038222) as a reference genome. We annotated the chloroplast genome with the software DOGMA (Wyman et al. 2004), and then corrected the results using Geneious 8.0.2 (Campos et al. 2016) and Sequin 15.50 (http://www.ncbi.nlm.nih.gov/Sequin/).

The complete chloroplast genome sequence of *S. bicapsularis* (GenBank number: MN873576) was characterized from Illumina pair-end sequencing. The chloroplast genome of *S. bicapsularis* was 161,056 bp in length, containing a large single-copy region (LSC) of 90,416 bp, a small single-copy region (SSC) of 18,538 bp, and two inverted repeat (IR) regions of 26,051 bp. The overall GC content is 36.20%, while the corresponding values of the LSC, SSC, and IR regions are 64.5%, 69.4%, and 60.2%, respectively. The genome contains 129 complete genes, including8 rRNAs, 37 tRNAs and 84 protein-coding genes. A phylogenetic analysis showed that *S.bicapsularis* and *S. occidentalis*, which belong to the group *Cassia*, share the closed relationship. The analysis of the cp genome of *S.bicapsularis* provides crucial genetic information for further studies of this precious species and the taxonomy, phylogenetics and evolution of *Cassia*.

We used the complete chloroplast genomes sequence of *S. bicapsularis* and 9 other related species and *Brassica napus* and *Arabidopsis thaliana* as an outgroup to construct the phylogenetic tree. The 10 chloroplast genome sequences were aligned with MAFFT (Katoh and Standley 2013), and then the neighbour-joining tree was constructed by MEGA 7.0 (Kumar et al. 2016). The results confirmed that *S.bicapsularis* was clustered with *S. occidentalis* (Figure 1).

**Figure 1.**
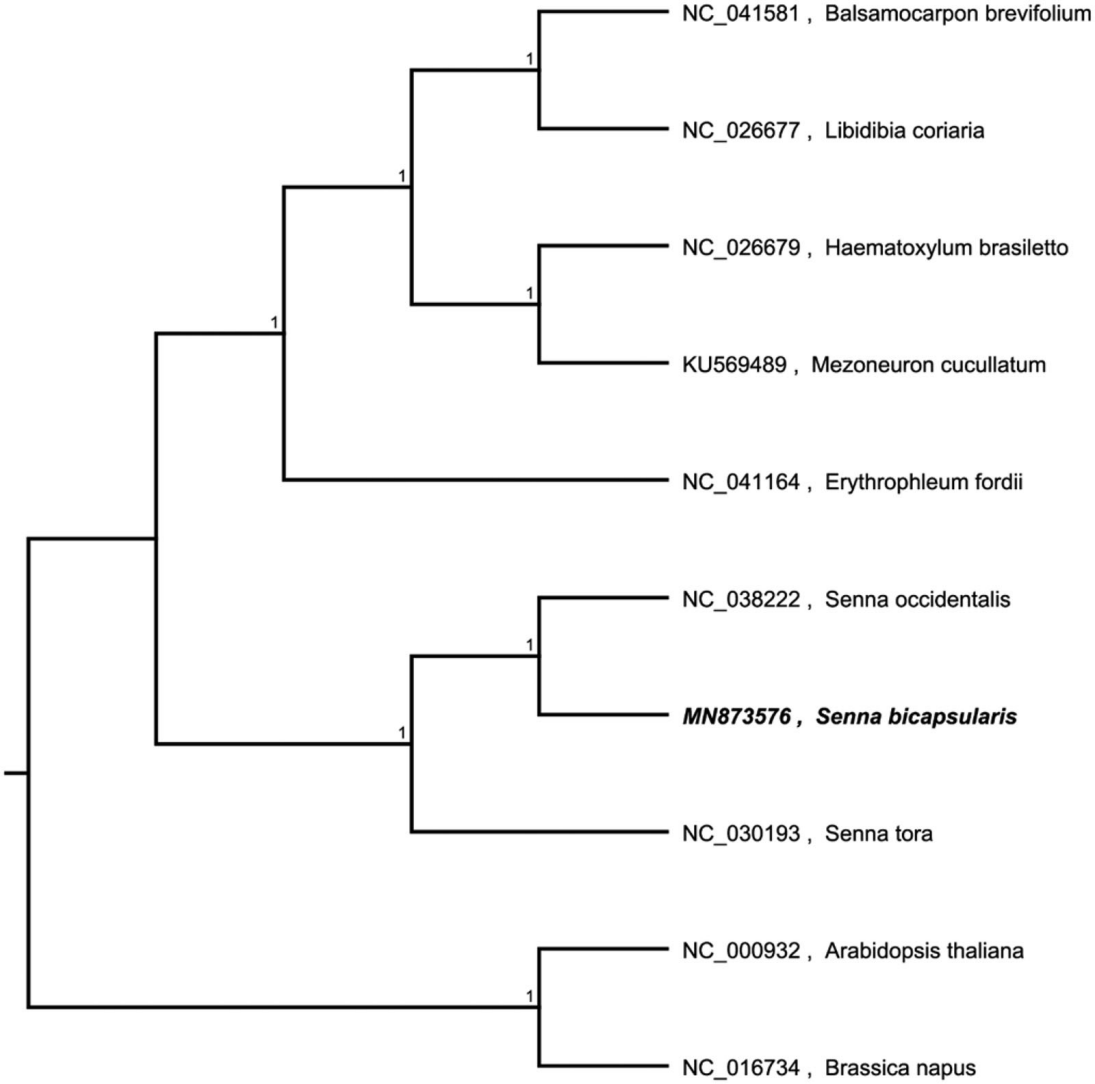
Neighbour-joining (NJ) analysis of *S. bicapsularis* and other related species based on the complete chloroplast genome sequence. Genbank accession numbers included in the figure.

## Data Availability

The GenBank accession number for the cp genome sequence of *S. bicapsularis* is MN873576 and the DOI is https://www.ncbi.nlm.nih.gov/nuccore/MN873576.
